# Observation of charged droplets from electrospray ionization (ESI) plumes in API mass spectrometers

**DOI:** 10.1007/s00216-021-03452-y

**Published:** 2021-07-02

**Authors:** Clara Markert, Marco Thinius, Laura Lehmann, Chris Heintz, Florian Stappert, Walter Wissdorf, Hendrik Kersten, Thorsten Benter, Bradley B. Schneider, Thomas R. Covey

**Affiliations:** 1 Institute for Pure and Applied Mass Spectrometry, Physical and Theoretical Chemistry, University of Wuppertal, 42119 Wuppertal, Germany; 2 SCIEX, 71 Four Valley Drive, Concord, ON L4K 4V8 Canada

**Keywords:** Electrospray ionization, Charged droplets detection, Droplet aspiration, Instrument contamination

## Abstract

Electrospray ionization (ESI) generates bare analyte ions from charged droplets, which result from spraying a liquid in a strong electric field. Experimental observations available in the literature suggest that at least a significant fraction of the initially generated droplets remain large, have long lifetimes, and can thus aspirate into the inlet system of an atmospheric pressure ionization mass spectrometer (API-MS). We report on the observation of fragment signatures from charged droplets penetrating deeply the vacuum stages of three commercial mass spectrometer systems with largely different ion source and spray configurations. Charged droplets can pass through the ion source and pressure reduction stages and even into the mass analyzer region. Since droplet signatures were found in all investigated instruments, the incorporation of charged droplets is considered a general phenomenon occurring with common spray conditions in ESI sources.

**Supplementary Information:**

The online version contains supplementary material available at 10.1007/s00216-021-03452-y.

## Introduction

Electrospray ionization (ESI) [1–3] is one of the most frequently applied ionization methods in mass spectrometry (MS) [4–7]. Essentially, an electrospray ion source consists of a glass or metal capillary in a strong electric field gradient. Liquid analyte solution is pumped or aspirated into the capillary. Charge separation processes in the liquid driven by the present electric field lead to the formation of a spray of charged droplets from the ESI capillary [6]. These charged droplets undergo a temporal evolution: Evaporation of neutral molecules, primarily solvents, leads to droplet shrinkage. Thus, the charge density due to the ions present in the droplet increases. If the resulting electric field in the droplet is high enough to overcome the surface tension of the liquid, often described as *Rayleigh limit*, fission occurs and the droplet disintegrates [8, 9]. These processes generally repeat multiple times. Eventually, very small and highly charged droplets with a radius of a few nanometers are formed from which gas phase ions are generated by different proposed mechanisms [1, 10–16]. The generation and evolution of the droplets have been studied extensively [8, 9, 17–25]. However, the experimentally observed initial droplet sizes often differ significantly from the size of first-generation droplets in the temporal evolution of fission events proposed in textbook literature [4, 6, 7] and older review articles [26, 27]. This proposed temporal evolution model starts with droplet sizes around 1–1.5 μm and assumes total droplet lifetimes well below 1 ms.

This is in disagreement to reported experimental results. Significantly larger and much longer lived droplets are experimentally found, mostly by direct optical detection of droplets with methods such as phase Doppler anemometry. Some exemplary results extracted from the literature are summarized in Table 1.

**Table 1:** Experimentally observed sizes of droplets generated in electrosprays

Analyte/solvent	Observed droplet size	Reference
Pure solvents (water, acetonitrile, *n*-heptane, *n*-octane, *p*-xylene)	25–35 μm with an observed droplet lifetime of 200–400 ms	Smith et al. [19] Grimm et al. [20]
Heptane	Up to 100 μm with 466 μL/min liquid flow rate, 5 μm with 4 μL/min liquid flow rate	Gomez and Tang [8]
Fluorescent dyes in acetonitrile	6 μm in positive mode, 20 μm in negative mode	Wortmann et al. [22]
Water/methanol solution with desorption electrospray ionization (DESI) emitter	Up to 10 μm with 2 μL/min liquid flow rate	Venter et al. [21]
Fluorescent dye in 20% methanol/water mixture	2 μm with 50 μL/min liquid flow rate	Girod et al. [24]

The results reported from diverse experimental setups described in the literature show a heterogenous and inconclusive picture. However, on average, the experimentally observed droplets are significantly larger and longer lived as the assumed initial particles in the proposed droplet evolution models. Cole gives an overview of some additional experimentally observed droplet sizes [6].

Experiments with charge detection mass spectrometry systems [28, 29] underline that large droplets generated by ESI can be transferred into the analyzer region of the used specialized instruments which allows analysis of the mass and charge distribution of such large droplet systems.

The diverging and sometimes even contradicting experimental results can be rationalized when considering the high complexity of the electrohydrodynamic processes occurring close to the capillary tip, which are leading to the generation of charged droplets. The electrospray process exhibits a variety of spray modes with different spray characteristics [30–32], which lead to a very broad range of initial droplet sizes [18, 33]. The spray mode depends strongly on the operation parameters, e.g., liquid flow rate, electric conductivity of the sprayed liquid or spray voltage. There is currently no comprehensive model, which sufficiently describes all possible spray modes.

In contrast, for the *cone jet* mode, which represents a particular spray mode of an undisturbed electrospray, a comprehensive model is available in the literature [34–39]. This mode is characterized by the formation of a Taylor cone and a fine jet emerging from the cone tip. The assumed initial droplet sizes in the proposed droplet evolution [9, 27] are derived from calculations of the liquid jet diameter in this cone jet mode [31, 40]. The required conditions for establishing an ideal, undisturbed, cone jet mode are more likely to be found in nano-electrospray (nESI) [5] sources. Peschke et al. [41] report a droplet evolution with an even smaller initial droplet size of 0.15 μm for nESI, which is also compiled in a review article by Kebarle and Verkerk [9]. Olumee et al. report a wide droplet size distribution with droplet sizes significantly below 1 μm [42], which is in turn referenced by Peschke to support the droplet evolution sequence for nESI. However, the *average* initial droplet size in these experiments was 1.5 μm using an electrically highly conductive liquid. Remarkably, it was observed that droplets from highly conductive solutions even grew larger with increasing distance from the ESI emitter.

A majority of electrospray sources employed for chemical analysis however are not of the nano-spray type. Common “high-flow” electrospray sources use large capillary diameters, high liquid flow rates, asymmetric electric fields, and strong assisting nebulizing gas flows which are injected coaxially to the ESI capillary in addition to sometimes extensive heating [5, 43, 44]. It thus appears to be unlikely that commercial high-flow electrospray ion sources typically generate initial droplets of 1 μm diameter, but rather a vast variety of initial droplet size distributions depending on the actual operating conditions of the ion source. The diverging experimental results mentioned above strongly support this notion. The experimental results reported suggest that the actual average initial droplet size in high-flow ion sources is significantly larger than 1 μm and the average lifetimes of the primary droplets are generally much longer than 1 ms.

It is concluded that highly charged droplets generated by electrospray in commercial ESI sources may well reach the MS inlet caused by the long droplet lifetimes. The time required by droplets to reach the MS inlet is readily estimated:

The terminal drift velocity *v*_*d*_ of charged particles moving in a gas within an electric field *E* is reached when the drag force *F*_*d*_ and the Coulomb force from the field *F*_*c*_ are in equilibrium:

1 $$ {F}_d={F}_c $$

The Coulomb force is the product of the droplet charge *q* and electric field E [45]:

2 $$ {F}_c=q\ E $$

The viscous drag force acting on a sphere with radius *R* moving with velocity *v* through a fluid with dynamic viscosity *μ* is estimated using Stokes law [46]:

3 $$ {F}_d=6\ \pi\ \mu\ R\ v $$

The drag force is affected by non-continuum effects for very small particles. This is considered with the Cunningham slip correction factor *C* [47]:

4 $$ C=1+\frac{2\ \lambda }{d}\left({A}_1+{A}_2\ \mathit{\exp}\left(\frac{-{A}_3\ d}{\lambda}\right)\ \right) $$

Here, *λ* is the mean free path of the background gas particles and *d* is the diameter of the charged particles. *A*_1_, *A*_2_, and *A*_3_ are empirical factors. For air, the numerical values are as follows: *A*_1_ = 1.257, *A*_2_ = 0.4, and *A*_3_ = 1.1 [47]. The calculated drag force is divided by *C*, yielding the actual drag force on a small particle.

Solving the force balance in Eq. (1) for *v* gives the estimated drift velocity:

5 $$ {v}_d=\frac{qEC}{6\pi \mu R} $$

The drift velocity is also described by the *electrical mobility K* of a charged particle [48]:

6 $$ {v}_d=K\ E $$

and thus:

7 $$ K=\frac{qC}{6\pi \mu R} $$

Experimentally generated droplets can reach surprisingly high electrical mobilities: The charged water droplets examined by Smith et al. [19] are representative in terms of experimentally observed droplet sizes. They have an initial diameter of approximately 27 μm and an initial total charge of 5 · 10^6^ elementary charges. With these parameters, the estimated initial ion mobility for such droplets is 1.7 cm^2^ V^−1^ s^−1^, which is very similar to the mobility of “naked” small molecular ions: The proton bound water cluster H_3_O^+^ · 2H_2_O has a mobility of 2.1 cm^2^ V^−1^ s^−1^ [49]. The estimated droplet mobility is in qualitative accordance to direct experimental observations of the velocity of mixed methanol/2-methoxyethanol droplets in an electric field by Grimm and Beauchamp [23]: The field gradient in the reported experiments was 50 V/cm and the observed droplet speed was around 55 cm/s, which corresponds to a mobility of 1.1 cm^2^ V^−1^ s^−1^.

The transfer times of such droplets from the ESI capillary to the MS inlet thus become very short. Assuming an average electrical field of 1000 V/cm and a transfer distance from the ESI capillary to the MS inlet of 1.5 cm, the required transfer times are between 1.3 and 0.8 ms for K = 1.1 and 1.7 cm^2^ V^−1^ s^−1^. This in fact is much shorter than the experimentally determined lifetimes of the charged droplets. Numerical simulation of a nano-ESI source further indicates transfer times of few milliseconds for ions with similar ion mobilities even with a substantial counter gas flow [50].

Since the electric mobilities of droplets are similar to the mobility of small molecular ions, droplets and ions will also have similar trajectories in the viscous environment of an atmospheric pressure high-flow ESI source. Therefore, charged ESI droplets are probably aspirated to a large extent into the vacuum system of MS. This notion is supported by experimental evidence available from the literature: Fomina et al. [51] investigated the transfer of droplets generated by an undisturbed cone jet electrospray into an MS interface. The droplets were comparably small and resembled closely the assumptions for the “ideal” droplet evolution sequence: The initial droplet size was around 1 μm and the droplet charge was around 10^4^ elementary charges, which results in a comparably low ion mobility of roughly 0.1 cm^2^ V^−1^ s^−1^, according to Eq. (7). Even in this case, the experimental results show that a large faction (up to 90%) of the total ion current enters the MS as charged droplets, which in turn survive the passage through inlet nozzles or inlet capillaries.

This situation leads to potentially adverse effects for the instrument operation: Large amounts of charged droplets aspirated into an MS result in large amounts of liquid solvent being deposited in the inlet system of the instrument. This leads to significant contamination of surfaces and ion optical elements in the first vacuum stages of an instrument, which results in increased maintenance times. This effect was demonstrated experimentally by Kang et al. [52]. In addition, incorporated droplets can induce complex physico-chemical processes regarding the analyte ionization pathway:

The detailed dynamics (e.g., trajectories, internal temperatures) of charged droplets in the electrical field and the pressure gradients within an MS inlet stage are currently unknown. Since the final release of analyte ions from incorporated droplets and nano-droplets potentially occurs deep inside the inlet system, the state of the droplets at this point in time and thus the detailed dynamics of the ion release is unclear as well. In addition, evaporating charged droplets can release a large amount of neutral solvents and similar components of the sprayed solution into the MS inlet stage which can interact with analyte ions, e.g., by clustering reactions [53–55]. Finally, the droplets may transport a large amount of charge into the MS system, which is however not visible in the recorded mass spectra. This could lead to unexpectedly high amounts of space charge in ion focusing and ion trapping elements, resulting in deteriorated performance. The aspiration of charged droplets potentially affects the analytical performance of instruments, even in cases when they do not reach the detector and are thus not directly visible in the recorded mass spectra.

It remains currently unclear how common the aspiration of charged droplets into the different stages of a mass spectrometer is, when using commercial ESI sources. The direct observations of droplets in such ion sources with optical methods as for example Doppler anemometry or micrograph flash photography represent high experimental efforts. However, Kang et al. reported an indirect but much more feasible approach to detect charged droplets and large clusters penetrating deeply a triple quadrupole MS system [52]. The first quadrupole (Q1) of the instrument was used in RF-only mode in these experiments. Q1 acts as a high-pass filter in this case, rejecting all particles with a mass-to-charge ratio (m/z) below the low mass cutoff (LMCO) of the quadrupole [56]. The subsequent collision cell and the third quadrupole of the instrument allow fragmentation of large particles, which have passed Q1, and mass analysis of the fragments of such particles, respectively. Similar indirect signatures of large charged aggregates and/or droplets penetrating the ion inlet stage are also detected with different experimental setups, e.g., using an early ESI interface with a heated inlet capillary [57].

To investigate how common droplet aspiration using commercial ion source/interface designs is, we searched for experimental evidence for charged droplets penetrating the inlet stages of three commercial API-mass spectrometers (a triple quadrupole, a quadrupole ion trap, and a quadrupole-ToF instrument) under various operational conditions.

Throughout this manuscript, we use the term “charged droplet/droplet fragments” for all multiply charged systems of a substantial size (at least a few nanometers in diameter) consisting of sprayed liquid originating from the spray plume.

## Methods

### Instruments

Three instruments equipped with largely different inlet systems were employed (cf. to Fig. S1 in Supplementary Information (ESM) for schematic depictions of the different ion paths in each instrument). The main focus was on a SCIEX Triple Quad 6500 System with an IonDrive Turbo V ion source, which was operated with a TurbolonSpray ESI probe (SCIEX, Ontario, Canada). The inlet of the MS is an orifice downstream of a curtain plate leading directly into the first focusing quadrupole (“Q-Jet”). It is noted that the SCIEX system has no inlet capillary. The temperature in the ion source is regulated by heaters. The spray needle was operated with a typical voltage of 5.5 kV, if not noted otherwise. The analyte solution was infused with the instruments’ internal syringe pump; the flow rate was set to 7 μL/min, if not noted otherwise. In consultation with the manufacturer, the system was operated in a special scan mode, which is usually not accessible via the instrument control software. In this “droplet scan” mode, the first mass selective quadrupole (Q1) is operated as a high-pass filter in true RF-only mode [52].

Droplets with m/z higher than the low mass cutoff (LMCO) m/z are transmitted through Q1 and are subsequently fragmented in the collision cell (which is also a quadrupole and is thus denoted as Q2) via collision-induced dissociation (CID). The background gas pressure of the nitrogen gas in the collision cell can be adjusted but is not measured directly by the instrument. The corresponding control software parameter (CAD) was set to CAD = 6 for most experiments, which corresponds to a pressure of approximately 5∙10^−3^ mbar in the collision cell.

Ions are accelerated into the collision cell by a potential difference between the Q0 region, in which the ions are focused, and the Q2 (“collision voltage”). This potential difference determines the kinetic energy of the analyzed ions and thus the collision energy in the fragmentation process. In the third quadrupole (Q3), a normal m/z scan is done, which allows recording the fragment spectrum.

This instrument has a high mass mode, which allows mass-scanning up to m/z = 2000, corresponding to a LMCO of approximately m/z = 1550, and a low mass mode with a maximum mass range of m/z = 1250 and a LMCO of about m/z = 990.

To investigate the transmission of charged droplets/droplet fragments into a largely different instrument, ESI experiments were performed with a Bruker amaZon ETD quadrupole ion trap (QIT). The instrument was fitted with an Apollo Ion Source (Bruker Daltonics, Bremen, Germany). Furthermore, it is equipped with an off-axis inlet capillary system as first pressure reduction system (instead of an orifice) and features a dual ion funnel assembly as inlet stage. The system is able to perform MS^n^ experiments: Ions of a selected mass range can be isolated and fragmented within the QIT. The resulting fragment ions can be mass analyzed or a subsequent stage of isolation and fragmentation can be executed. The voltage on the spray shield was set to −4.5 kV for positive mode. In a set of experiments, the ion trapping window was set to 2500 m/z with a width of 100 m/z. The storage time was varied between 40 and 1000 ms.

A third instrument was used to gather additional information about any charged droplets/droplet fragments detected: A solution of thermometer ions was analyzed in an Agilent 6538 UHD Q-ToF (Agilent, Waldbronn, Germany). This instrument has a similar capillary entrance system as the Bruker QIT, but without ion funnels for focusing and transporting ions; here the metallized capillary exit cap and a subsequent skimmer act as focusing elements. This Q-ToF has a much wider m/z range, reaching m/z = 10,000. The ESI voltage was set to 2.9 kV with a liquid flow rate of 8 μL/min. The mass selective quadrupole of the instrument was set to 2500 m/z and the DC was switched off, which again creates an RF high pass filter. Similarly to the SCIEX triple-quadrupole instrument, the ions pass a collision cell quadrupole and are finally transferred into the ToF mass analyzer. This allows running the droplet fragmentation experiments similarly to the triple-quadrupole instrument, but with a much wider mass range.

Due to limitations of the control software of the Q-ToF instrument, no acquisition mode (i.e., storable) spectra with the first quadrupole in RF-only mode and different collision energies in the collision cell could be recorded. However, the software shows the live mass spectra in tune mode, in which the DC on the first quadrupole can be switched off. We thus recorded screenshots of the mass spectrum window in tune mode. These are shown in the ESM.

### Chemicals

Para-substituted benzylpyridinium ions were used as analytes for experiments with the triple-quadrupole and ion trap instruments [58, 59]. They are referred to as *thermometer ions* due to their well-known fragmentation behavior depending on their internal energy. The compounds were synthesized in one-pot synthesis from derivatized benzyl bromide (96–98% purity, obtained from Sigma-Aldrich), with equimolar amounts of dry pyridine (⩾ 99% purity, obtained from J.T. Baker) and partially acetonitrile (HPLC-gradient grade, obtained from VWR chemicals) as solvent. After the reaction had been completed under constant stirring, the benzylpyridinium species were purified by re-crystallization from ethanol (96% purity, obtained from Sigma-Aldrich) or diethyl-ether (⩾ 98% purity, obtained from Merck KGaA). This synthesis is based on the experimental specification by Katritzky et al. [60], but has been modified in some steps: Acetonitrile (HPLC-gradient grade, obtained from VWR chemicals) and water (ultrapure water from water treatment system Milli-Q Reference) were used as solvent. For better spraying conditions, 0.1 %V formic acid (~ 98% purity, obtained from Fluka Analytical) was added to the solution. A solution of 10 μmol/L of each thermometer molecule species in 1:1 mixture of water/acetonitrile was used in the experiments.

The m/z of the precursor and fragment ions are shown in Table 2. A second set of experiments was done with reserpine (≥ 99% purity, obtained from Sigma-Aldrich) in a 1:1 mixture of isopropanol (HPLC grade, obtained from Fisher Chemical)/water and 0.1% formic acid (≥ 98% purity, obtained from Sigma-Aldrich) added. The solution had a concentration of 0.8 μmol/L.

**Table 2:** Nominal masses of benzylpyridinium (p) thermometer ions and their main fragment species [61]

Ions	m/z (precursor ion)	m/z (fragment ion)
*p*-CH3	184	105
*p*-F	188	109
*p*-Cl	204	125
*p*-CN	195	116
*p*-NO2	215	136

## Results and discussion

### Droplet data from triple-quadrupole MS (SCIEX 6500)

#### Benzylpyridinium thermometer ions

In the first set of experiments, the SCIEX Triple Quad 6500 system was run in a “droplet scan mode” by setting Q1 to RF-only mode as described in the “Methods” section. The LMCO was m/z 1550 in this set of experiments.

**Fig. 1:**
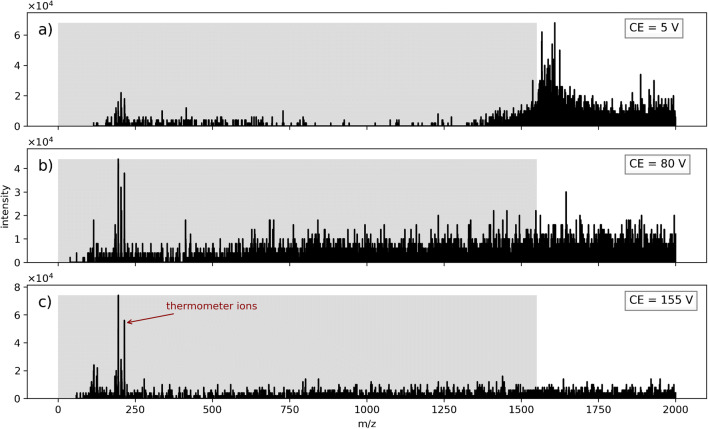
SCIEX Triple Quad 6500 instrument: Spectra recorded in droplet scan mode with thermometer ions sprayed from a water/acetonitrile solution at different collision voltages. Collision gas parameter (CAD) was set to 6. Liquid flow was set to 7 μL/min. The m/z region filtered out in Q1 is indicated by the shaded areas

Figure 1 shows the mass spectra of the thermometer ions in a 1:1 water/acetonitrile solution. At a collision voltage setting of 5 V (Fig. 1a), an abundant and wide signal distribution is discernible above the LMCO. Analyte signals are detected in the range around m/z 200 with moderate intensities. Additional unassigned signals below the LMCO are present with low intensities. These low m/z ions are generated *downstream* of Q1, since this mass range is rejected from the ion beam by Q1 acting as high-pass filter. In Fig. 1b, the collision voltage is increased to 80 V. The signals above the LMCO decrease, while the intensity of the analyte signals, most pronounced between m/z 184–215, and the signal intensity of the entire mass region below the cutoff increase. The LMCO is at this point no longer discernible in the mass spectrum. When the collision voltage is further increased to 155 V, the signals above the LMCO almost vanish and the primary analyte signals at m/z 204 (precursor species *p*-Cl) (cf. Table 2) and m/z 215 (precursor species *p*-NO_2_) almost double in intensity. The change in the observed signal pattern is continuous: There are no abrupt changes in the spectrum pattern when the collision voltage is varied.

The high ion current *above* the LMCO in the spectrum at low collision energy (Fig. 1a) and the occurrence of analyte signals *below* the LMCO indicate that substantial amounts of large charged aggregates enter the system, although countermeasures (e.g., curtain gas flow) within the ion source were operated regularly in this experiment. Notably, the variation of the curtain gas flow does not have a significant impact on the charged droplets/droplet fragments in the spectra (cf. ESM Fig. S2). Considering the experimental information from the literature described in the Introduction we attribute the observed signals to charged droplets/droplet fragments of the sprayed fluid with initial diameters well above 1 μm. This is underlined by the occurrence of analyte signals shown in Fig. 1b and c: The observed bare analyte ions have to be transported through Q1 within charged droplets or large droplet fragments. These particles penetrate the collision cell (Q2) and interact with the background/collision gas under the influence of the collision voltage. The increase of collision energy leads to a stronger extent of collision induced dissociation/fragmentation inside the collision cell. This in turn shifts the droplet sizes to smaller values by fragmentation and leads eventually to the generation of bare analyte ions. The released analyte ions are detected subsequently in a Q3 scan, which explains the larger abundance of bare analyte ions in the spectra with high collision voltage present. Furthermore, the analyte ions released from the droplet phase fragment as well, depending on the collision energy settings. The signals of the main fragments of the thermometer ions appear around m/z 100 (cf. Table 2).

Droplets penetrating deeply the MS system conclusively explain the results from the first set of measurements. A lower limit of the droplet size is estimated by transforming the equation for the number of charges in a droplet at the Rayleigh limit [6, 61]:

8 $$ Q=\kern0.5em 8\pi {\left({\epsilon}_0\gamma {r}^3\right)}^{1/2} $$

With the number of charges *Q*, the electrical permittivity ε_0_, the surface tension γ, and the radius of the droplet r. The mass of a spherical droplet is derived from the density of the liquid forming the droplet and the corresponding radius. Thus, the radius determines the mass and the critical charge of the droplets at their Rayleigh limit, which allows calculation of the critical m/z, as presented in Table 3.

**Table 3:** Estimated radius r of critical spherical droplets at the Rayleigh limit

	Surface tension (mN/m)	Density (g/cm^3^)	r for m/z = 990 (nm)	r for m/z = 1550 (nm)	r for m/z = 2000 (nm)
Water	72.8	1.00	1.3	1.8	2.0
Acetonitrile	29.0	0.79	1.2	1.6	1.9
Methanol	22.7	0.79	1.1	1.4	1.7

This estimate shows that droplets with m/z between 1550 and 2000, which is well in the mass range above the LMCO visible in the triple-quadrupole instrument, consisting of a water/acetonitrile mixture, should have a minimal radius above the critical limit of 2 nm. Charged fragments of even larger droplets do fall also in this size regime and can contribute to the observed ion current above the LMCO.

#### Collision gas pressure variation

In a second set of experiments, the droplet scan was repeated in the low mass mode of the SCIEX instrument. The low mass mode provides an analyzer mass range of up to m/z 1250, with a higher resolution than the high mass mode. The LMCO shifts down to approximately m/z 990. The calculated size of detectable droplets (cf. Eq. (8)) shrinks to about 1–1.25 nm in radius (cf. Table 3).

The pressure in the collision cell is determined by the CAD parameter in the control software of the instrument. The pressure is ranging between 1 × 10^−3^ and 9 × 10^−3^ mbar; the default setting for the CAD parameter is 6, which corresponds to approximately 5 × 10^−3^ mbar.

**Fig. 2:**
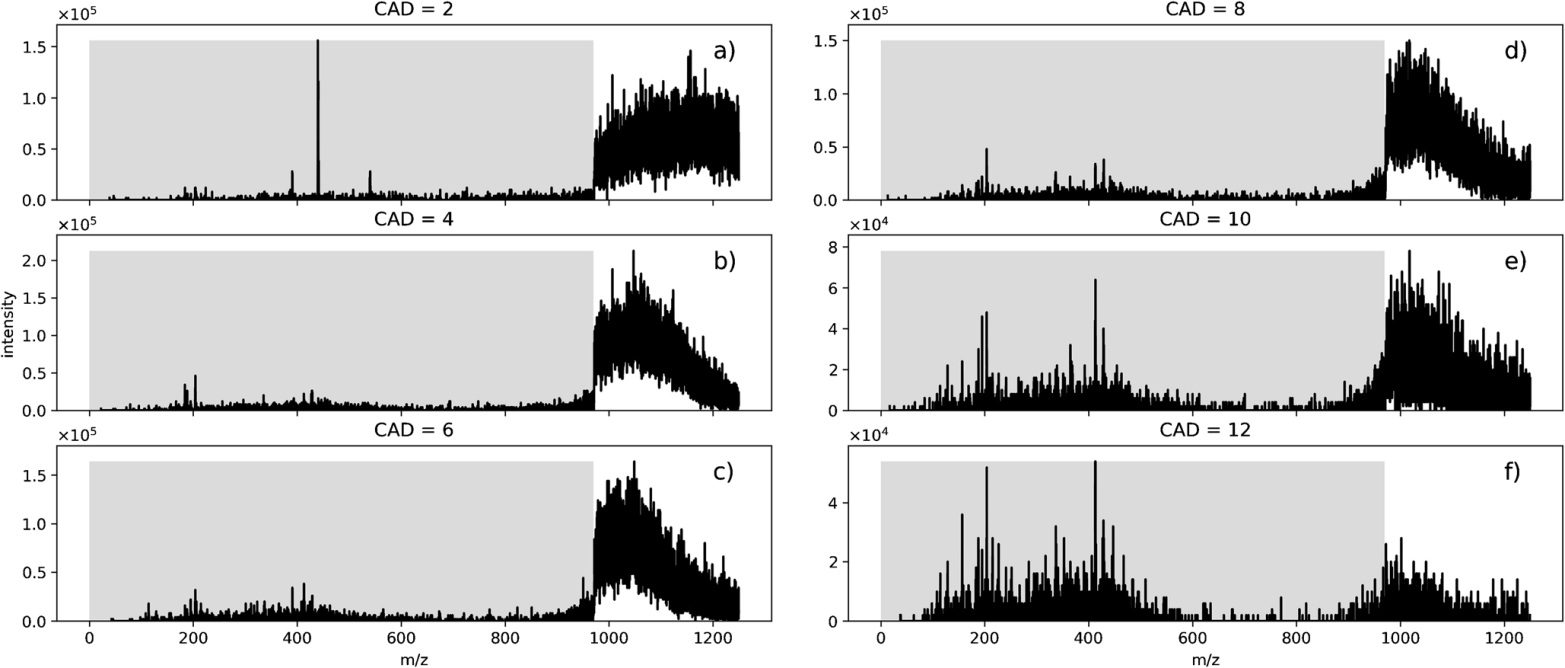
SCIEX Triple Quad 6500: Variation of gas pressure (CAD parameter) in the collision cell at a fixed collision energy of 12 V. Measurement of benzylpyridinium thermometer ions sprayed from water/acetonitrile solution in low mass mode. Liquid flow was set to 7 μL/min. The m/z region filtered out in Q1 is indicated by the shaded areas

Figure 2 presents the result of the variation of the collision gas pressure, at a fixed collision voltage of 12 V. As expected, the pressure variation demonstrates that the fragmentation process of the droplets is determined by not only the average collision energy but also the collision frequency, which are both governed by the collision gas pressure. When the CAD parameter is increased, the abundance of signals above m/z 990 is shifted to smaller m/z, which indicates the disintegration of the droplets.

**Fig. 3:**
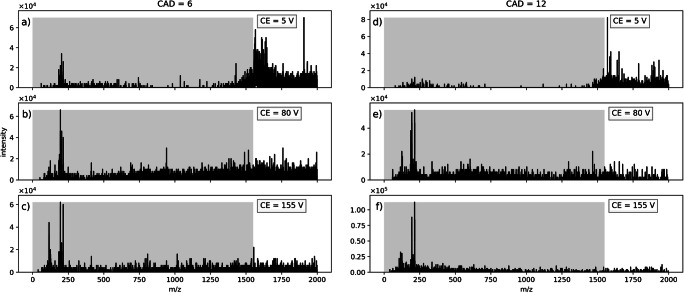
SCIEX Triple Quad 6500: Variation of gas pressure in the collision cell at different collision energies. Measurement of benzylpyridinium thermometer ions sprayed from water/acetonitrile solution in high mass mode. Collision gas parameter (CAD) was set to 6 (left) and 12 (right). Liquid flow was set to 10 μL/min. The m/z region filtered out in Q1 is indicated by the shaded areas

With decreasing collision gas pressure, and therefore less gas collisions, ions above the LMCO begin to pass through the collision cell unaffected. As a consequence, a sharp signal step occurs at the LMCO due to the high-pass filtering of Q1, which is “blurred” at higher CAD values. The sharp signal cutoff at the LMCO boundary is much more apparent in the low mass mode as it was in the high mass mode (cf. Figs. 2 and 3), but it occurs reproducibly with very low collision gas pressures in both operation modes (not shown here for the high mass operation mode). Although the shape of the continuous signal above the LMCO with smaller collision energies is highly reproducible between two experiments in one of the operation modes, the selection of an operation mode appears to have a significant effect on the droplet fragmentation and potentially the transfer efficiency through the collision cell. The cause of this effect is speculated to be caused by changes of the DC voltage and RF amplitude configuration along the ion path when the operation mode is changed.

The variation of the collision gas pressure was also done in high mass mode with thermometer ions sprayed from water/acetonitrile solution. CAD settings of 6 (about 5 × 10^−3^ mbar cell pressure) and 12 (9 × 10^−3^ mbar cell pressure) are compared in Fig. 3 for different collision energies. Note that the liquid flow rate in Fig. 3a–c is higher (10 μL/min) than that in the set of spectra at the same CAD and comparable collision voltages shown in Fig. 1 (7 μL/min).

If the pressure in the collision cell is set to CAD = 12, the ion current above the LMCO is smaller (Fig. 3d) than at lower pressure. The overall intensity below the LMCO is reduced as well. At the default setting of CAD = 6 and a collision energy of 5 V, small analyte signals are visible at approximately m/z 200. When the collision energy is increased in the same increments as in the first set of experiments, the signals at a higher m/z decrease, similar to the spectra shown in Fig. 1. Due to the higher collision gas pressure in the cell, the charged droplet/droplet fragment signals above the LMCO decrease and the fragments in the spectrum Fig. 3e are on average smaller as in Fig. 3b. In the last spectrum (Fig. 3f), the signals of the unidentified fragments have vanished when compared to the signals of the liberated bare analyte.

The results obtained with the triple-quadrupole instrument provide substantial evidence of the presence of droplets in such a type of commercial instrument. The behavior of the droplets and their disintegration can be influenced by the applied collision energy and the background gas pressure in the collision cell.

The chemical composition of the sprayed solution is very likely an important contributing factor for the behavior of the droplets in the instrument. Differences are likely to be discernible in the mass spectra. To validate this hypothesis, the experiments were repeated with reserpine as analyte in a 1:1 mixture of isopropanol/water with the same settings of the droplet scan and the same collision energies.

#### Reserpine

Another set of experiments was performed to investigate the influence of different solvents used for spray generation on the droplet fragment spectra. The SCIEX triple-quadrupole instrument with the same system settings as described above was used, but with reserpine as analyte and isopropanol/water as solvent. As before, the protonated analyte reserpine at m/z 609 is filtered out by Q1 in high pass mode.

**Fig. 4:**
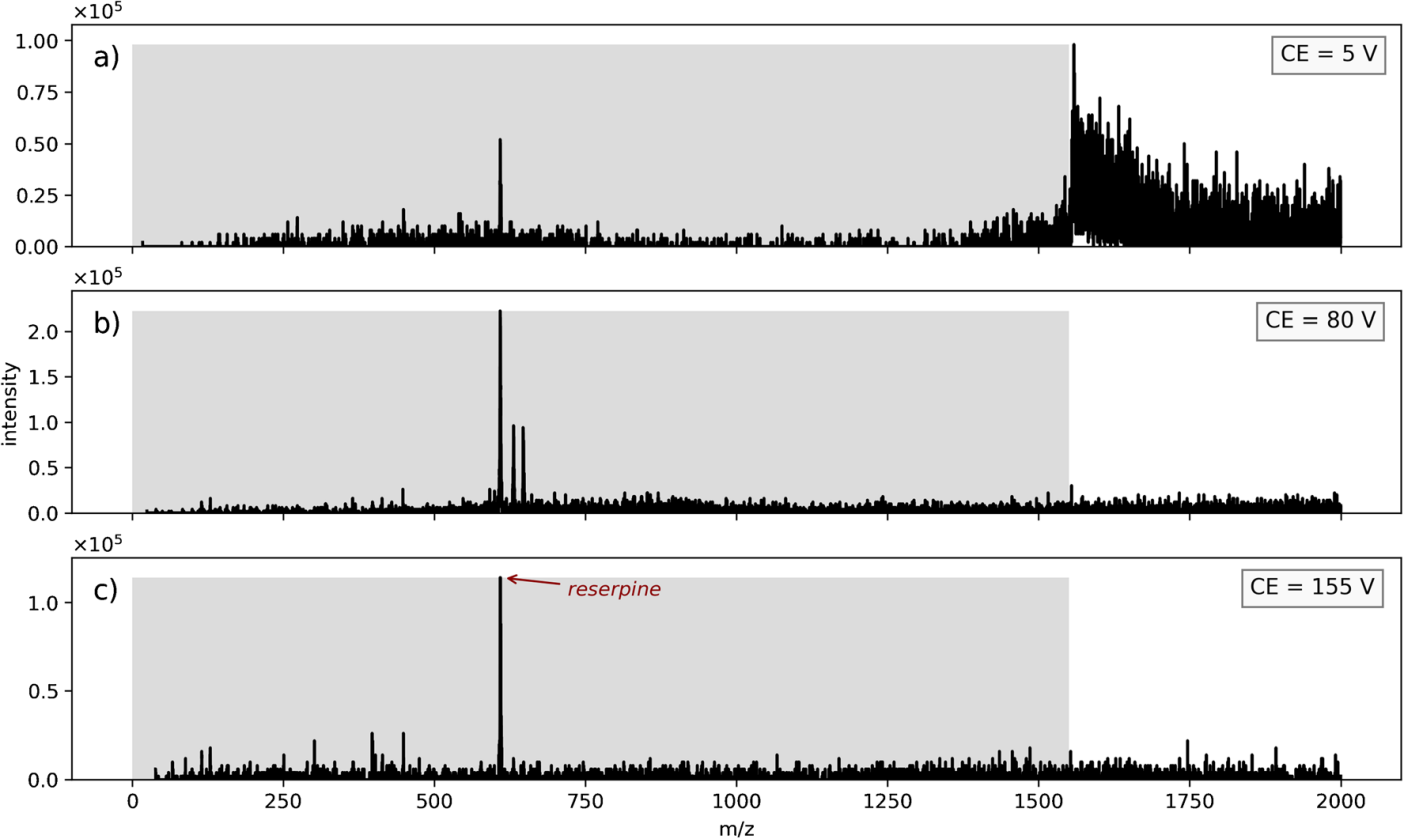
SCIEX Triple Quad 6500: Droplet scan with reserpine sprayed from isopropanol/water solution at different collision voltages. The m/z region filtered out in Q1 is indicated by the shaded areas

In comparison to the results with the thermometer ions, the overall appearance of the mass spectra is similar. However, there are notable differences in the signal shape above the LMCO: The thermometer ion solution shows a broad, nearly continuous signal, particularly in the region around the LMCO at 5 V collision voltage. In contrast, an abrupt signal drop at the LMCO boundary around 1550 m/z is discernible when spraying the reserpine solution. The much less pronounced “bleed-over” towards signals below the LMCO in Fig. 4a indicates less fragmentation at 5 V collision voltage with the reserpine solution. A possible explanation for this finding is the protic behavior of isopropanol as a solvent. It potentially leads to a higher stability of the charged droplets due to more pronounced hydrogen bond formation in comparison to droplets consisting of a mixture of acetonitrile and water [62]. Subsequently, this could lead to a different fragmentation pattern of the large primary droplets and eventually to the observed discontinuity in the fragment signals of the isopropanol/water droplets.

Nevertheless, the general appearance of the observed reserpine solution mass spectra is essentially similar to the findings observed with the thermometer ions: A wide continuous signal is found above the LMCO with low collision voltage (Fig. 4a) and, as seen above, a higher collision energy leads to the appearance of bare analyte ion signals, while the signal above the LMCO vanishes (Fig. 4b and c). The signal intensities do not change considerably with varying collision voltages, although the signals in spectra with a collision voltage of 80 V have a slightly higher intensity.

The comparison between the fragment spectra of the two investigated analyte/solvent systems thus strongly suggests that the choice of solvent has, among numerous other things, also a significant influence on the droplets population and thus the resulting fragment mass spectra.

### Bruker ion trap data

The mixture of thermometer ions dissolved in water/acetonitrile was also analyzed with a Bruker amaZon ETD quadrupole ion trap. In one set of experiments, ions around m/z 2500 were trapped, with the width of the trapping window set to ± 50 m/z. The source temperature was set to 50 °C; all other ion source parameters and ion transfer parameters (dry gas flow, ion funnel potentials, etc.) were within common operation conditions.

**Fig. 5:**
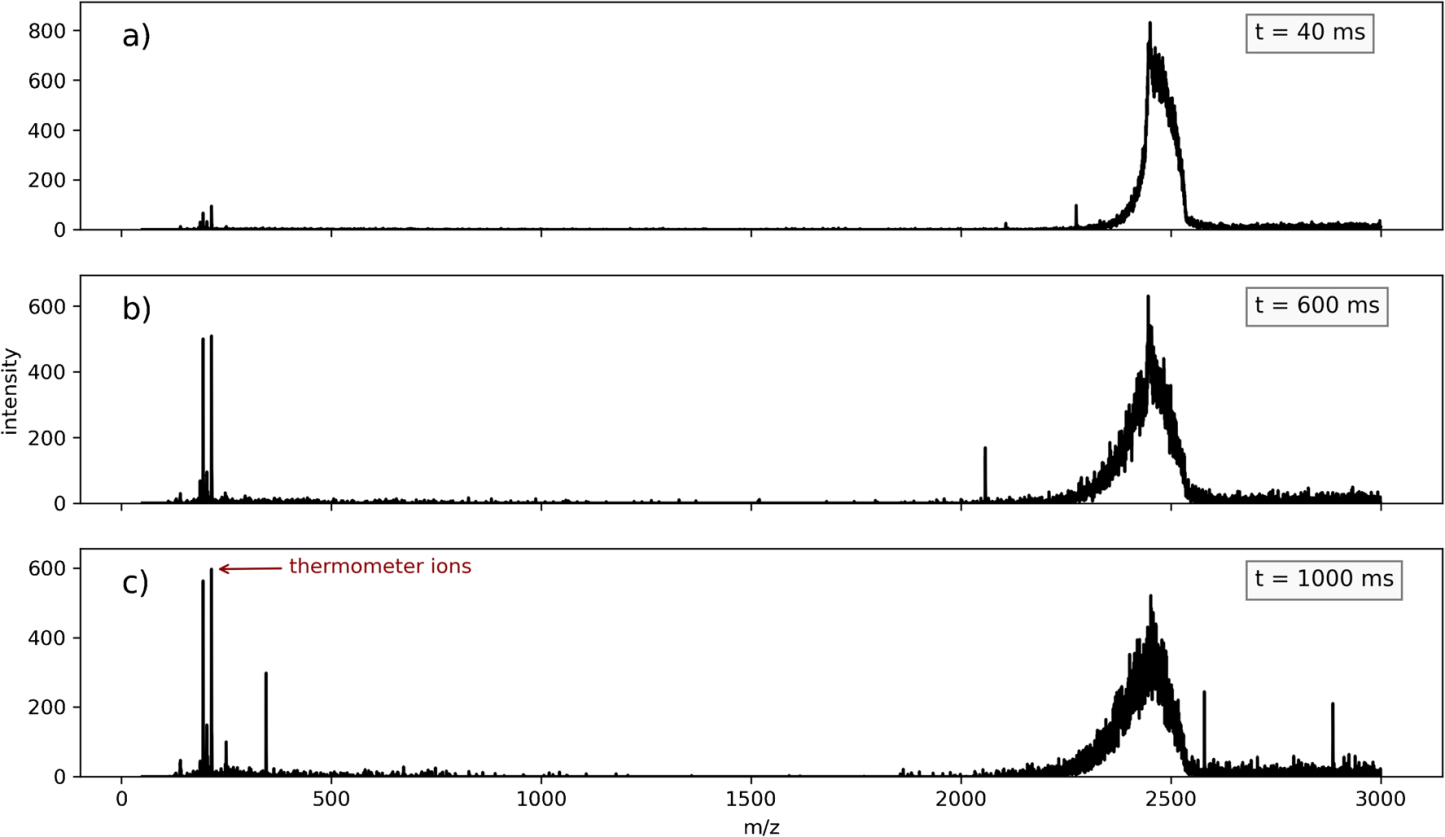
Spectra from benzylpyridinium in water/acetonitrile in a Bruker amaZon ETD ion trap. Measured at an ion source temperature of 50 °C for different storage times

The storage time within the trap prior to mass analysis was varied systematically. A very broad signal peak appears in the defined trapping window, which indicates that charged droplets/droplet fragments enter the mass analyzer region and can thus be directly detected with the ion trap instrument. With a storage time of 40 ms, only small analyte signals are visible (cf. Fig. 5a) and the signal peak in the trapping window around m/z 2500 is relatively narrow. When the storage time is increased to 600 ms, the signals of the analyte ions start to increase and the signal peak at m/z 2500 broadens unsymmetrically towards smaller m/z (cf. Fig. 5b), which implies that the droplets release bare analyte ions inside the trap caused by collision-induced droplet evaporation. With a storage time of 1000 ms, the signal at m/z 2500 is broadened even further and the bare analyte ion signals are clearly defined.

This remarkable result is even stronger evidence that droplets are penetrating the mass analyzer region of an instrument with a completely different entrance system from the SCIEX triple-quadrupole instrument. Recall that the Bruker QIT features an off-axis capillary inlet stage with two subsequent focusing RF ion funnels, whereas the SCIEX instrument has on-axis open nozzles and transfer quadrupole (Q-jet). Furthermore, the release of the charged analytes is influenced not only by the gas pressure and collision energy prevailing to them, but also potentially by the time the droplets are stored, as the evaporation of neutral particles from the droplets continues. The possible lifetimes of charged droplets/droplet fragments in the ion trap exceed 1 ms by far.

### Q-ToF data

The solution of thermometer ions in acetonitrile/water was analyzed with the Agilent Q-ToF. As described in the “Methods” section, acquisition-mode mass spectra with the first mass selective quadrupole in RF-only mode could not be recorded with the control software, but screenshots of the measurements in tune-mode are shown in the ESM. For the experiments, the DC applied to the quadrupole in front of the collision cell was turned off, resulting again in high-pass mass filter operation. The target range to be filtered out by the quadrupole was set to m/z 2500 and the collision voltage was ramped in three increments (0, 50, and 100 V).

With these settings, a broad signal peak appears around m/z 2000. The intensity of this structure in the spectrum with the collision voltage set to 0 V is considerable compared to the small signals of bare analytes visible (cf. ESM Fig. S3a). This structure of peaks is evidence of droplets passing the quadrupole since the bare analyte is filtered out. With the collision voltage raised to 50 V, the analyte peaks start to increase drastically. The peak structure around m/z 2000 remains visible. The analyte signals increase even more with the collision voltage set to 100 V (cf. ESM Fig. S3c). The peak structure at m/z 2000 is not as defined but still discernible. The analyte signals increase about 500% by solely ramping the collision voltage, which indicates that the droplets are releasing the analytes in the same manner as in the SCIEX triple quadrupole and the Bruker ion trap instrument.

## Conclusions

The experimental observation of droplet fragments in the investigated mass spectrometric systems demonstrates that large amounts of charged droplets are likely to be aspirated into the instruments and penetrate deeply the different vacuum stages. This aspiration was observed with all investigated instruments. The geometries of the ion source enclosures and ESI emitter assemblies, as well as the applied gas flows, are largely different in the three systems (cf. ESM Fig. S1). This holds true for the ion transfer stages to the analyzer region as well: (i) nozzle/skimmer + transfer quadrupole (Q-Jet, SCIEX triple quadrupole), (ii) off-axis capillary + dual ion funnel + transfer multipoles (Bruker amaZon QIT), and (iii) on-axis capillary + skimmer + transfer quadrupole (Agilent Q-ToF).

Therefore, significant droplet aspiration is likely a general phenomenon occurring when “high-flow” ESI sources are operated on commercial API instruments and not an edge case of a certain ion source configuration or design. The available experimental results from the literature unequivocally show the existence of large and long-living charged droplets/droplet fragments generated by ESI. This is in full accordance with our experimental findings. In future works, we will elucidate the fragmentation/evaporation process of these droplets in response to the variation of inlet and transfer parameters.

ESI is one of the most widely used ionization techniques and has thus high importance for analytical chemistry. The possible consequences of droplet aspiration into mass spectrometers are more diverse than just chemical noise in the resulting mass spectra: The contamination due to droplets penetrating the inlet stages would lead to increased maintenance and instrument down-time. Additionally, the dynamics of the proposed droplets in the instruments are currently unknown. Thus, the possible chemical and physical consequences of the presence of droplets and their fragments in MS inlet stages are also unknown. Besides possible complex direct chemical interactions between droplets and droplet constituents, secondary effects like space charge saturation could be relevant. Such effects would be particularly problematic in mobility pre-separation stages (DT-IMS, TWIMS, DMS, TIMS).

These results, along with the body of literature data, also strongly challenge the assumptions of the established quasi-equilibrium droplet evolution models [9, 25]. The latter imply the complete evaporation of the spray droplets and the generation of bare ions within the *ion source region*, which is in stark contrast to the experimental observations. However, the evolution models are not wrong per se; they still are highly valuable models for the processes occurring during the final stages of bare ion generation from charged droplets. However, for a significant fraction of the observed total *source* ion current generated, those final steps are taking place far downstream of the source, i.e., deep in the vacuum stages of the instrument.

This conclusion has a number of consequences: Droplets present in the vacuum stages of a mass spectrometer are generally exposed to completely different physical and chemical conditions as compared to the situation in the AP-ion source region; strong pressure and temperature drops in supersonic expansions of the background gas with shock phenomena, strong electric field gradients, and, due to the much lower collision frequency, increased electric acceleration of charged particles dominate the different vacuum stages of an API mass spectrometer [55]. This further complicates the droplet evaporation/fragmentation processes finally resulting in bare molecular ions, and much more so corresponding modeling attempts.

The comprehensive elucidation of the droplet dynamics in API mass spectrometers thus requires an interdisciplinary research program: Multiple experimental and modeling efforts along the ion path through the instruments are required to build a more complete picture of the ESI process. After all, ESI works in many, many cases beautifully well. A whole industry builds around it since about 5 decades. So why bother? Because terms as “ion suppression,” “unexpected results,” or “magic ionization” are making physical and theoretical chemists … restless.

## Supplementary information

ESM 1 (DOCX 620 kb)
