# Supplementary material for: Observation of charged droplets from electrospray ionization (ESI) plumes in API mass spectrometers
Source: Anal Bioanal Chem. 2021 Jul 2;413(22):5587–600. doi: 10.1007/s00216-021-03452-y (PMC8410725; doi:10.1007/s00216-021-03452-y)
Supplement: Supplementary file 1 — (DOCX 620 kb) [file 216_2021_3452_MOESM1_ESM.docx]

# **^Supplementary Information^**

**Fig. S1:** Schemes of the instruments used for the presented study


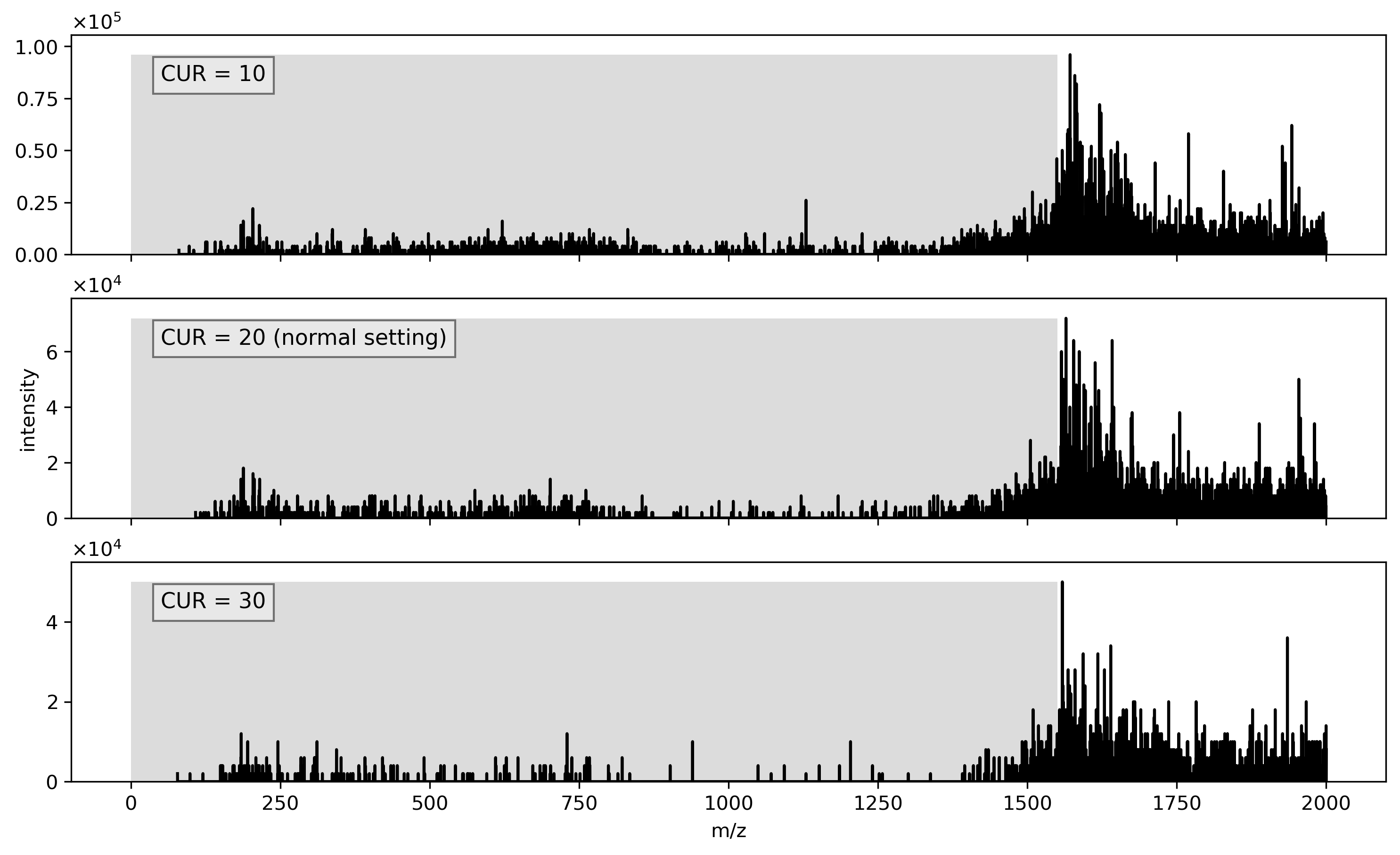


**Fig. S2:** Droplet Scan of thermometer ions in ACN / H_2_O with varying curtain gas flow.


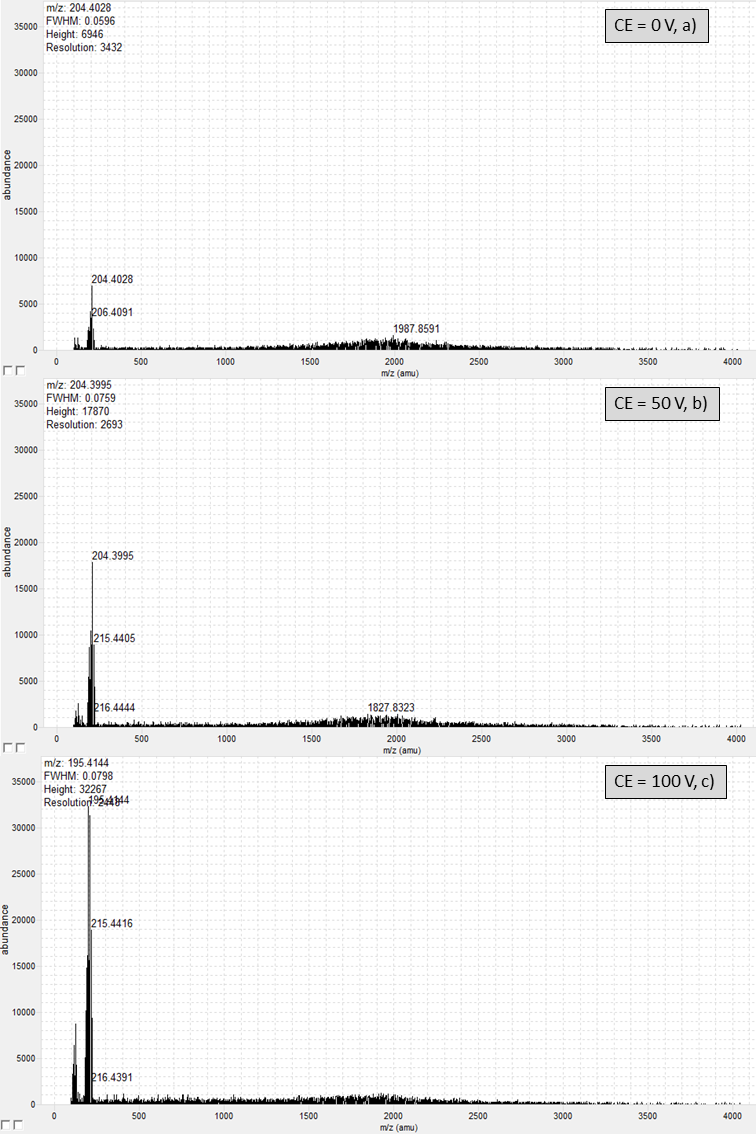


**Fig. S3**: Screenshots of the spectra measured in the tun- mode of the Agilent Q-ToF at different collision voltages applied.
